# Pelvic Organ Prolapse with an Emphasis on the Central Compartment: From Genetic Risk Factors and Biomarkers to Contemporary Sacropexy and Emerging Robotic Innovations

**DOI:** 10.3390/jcm15134967

**Published:** 2026-06-25

**Authors:** Michał Pomorski, Tomasz Fuchs, Anna Kryza-Ottou, Joanna Budny-Wińska, Jakub Śliwa, Adam Pomorski

**Affiliations:** 1 Department of Obstetrics and Gynecology, University Center of Obstetrics and Gynecology, Wroclaw Medical University, 50-556 Wroclaw, Poland; 2 Department of Chemical Biology, Faculty of Biotechnology, University of Wrocław, 50-383 Wrocław, Poland

**Keywords:** pelvic organ prolapse, sacrocolpopexy, robotic-assisted sacrocolpopexy, biomarkers, molecular basis of the disease

## Abstract

Apical pelvic organ prolapse (POP) is characterized by descent of the uterus or post-hysterectomy vaginal vault resulting from failure of level I pelvic support and represents a major contributor to pelvic floor dysfunction and recurrent prolapse surgery. Loss of apical support is frequently associated with anterior and posterior compartment defects, leading to vaginal bulge symptoms, pelvic pressure, urinary and bowel dysfunction, sexual dysfunction, and reduced quality of life. This narrative review summarizes current knowledge on POP, from molecular mechanisms and emerging biomarkers to contemporary surgical management, with particular emphasis on sacrocolpopexy and robotic-assisted approaches. A literature search of PubMed, Scopus, Google Scholar, and Consensus identified peer-reviewed studies published up to February 2026. Evidence demonstrates that POP has a multifactorial and polygenic background involving extracellular matrix remodeling, connective tissue integrity, smooth muscle dysfunction, and altered level of protein expression. Several candidate biomarkers, including single-nucleotide polymorphisms, circulating proteins, metabolites, and imaging-based parameters, show potential for risk prediction and earlier diagnosis, although routine clinical implementation remains limited. Sacrocolpopexy remains the gold standard for apical prolapse repair because of superior anatomical outcomes, low recurrence, and significant quality-of-life improvement. Laparoscopic and robotic-assisted sacrocolpopexy provide comparable efficacy with reduced blood loss, shorter hospitalization, and faster recovery. The objective success rate is usually over 90%. Complications are very rare and typically include mesh erosion in 2–4% of cases and the need for reoperation in 6% of cases. Our own experience shows that, for a group of surgeons, the learning curve for the laparoscopic approach reached a plateau after a total of 30 operations. Robotic platforms may facilitate complex pelvic dissection and shorten the learning curve, although higher procedural costs remain a major limitation.

## 1. Introduction

Apical pelvic organ prolapse (POP) represents the descent of the uterus, or post-hysterectomy vaginal vault due to failure of level I pelvic support [1]. Adequate apical support is critical to overall pelvic floor integrity, and loss of this support is frequently associated with anterior and posterior compartment prolapse, leading to significant pelvic floor dysfunction. Women with apical defects commonly experience a vaginal bulge sensation, pelvic pressure, urinary and bowel symptoms, and sexual dysfunction, all of which substantially impair quality of life. The terms apical, central and middle compartment, are used interchangeably in this review as they describe prolapse resulting from the DeLancey’s level I support failure. The lifetime risk of undergoing surgery for POP or urinary incontinence is estimated to be approximately 12.6–19%, with apical prolapse being a major contributor to surgical failure if not adequately addressed [2,3,4]. Epidemiologically established risk factors for apical defects include vaginal childbirth, advancing age, obesity, chronic increases in intra-abdominal pressure, and prior pelvic surgery [5]. More recently, genetics has been added as it shows a strong etiologic link to the development of POP, to which other factors may add or protect against the risk [6]. The incidence of vault prolapse following hysterectomy has been reported to be 0.5% [7]. Other reports state that the incidence of vaginal vault prolapse following hysterectomy varies from 1.8% when hysterectomy was performed due to non-prolapse related benign diseases to 11.6% when hysterectomy had been performed for genital prolapse [8].

Surgical management of middle compartment pelvic organ prolapse includes a wide range of abdominal and vaginal techniques aimed at restoring apical support while optimizing anatomical and functional outcomes. Abdominal approaches—such as colpo-, cervico-, or hysterosacropexy, lateral uteropexy, pectopexy, and anterior abdominal fixation—are most often performed using minimally invasive techniques and are designed to recreate apical suspension. However, only sacropexy recreates the physiological vaginal axis. The terms sacropexy, sacrocolpopexy (colposacropexy), cervicosacropexy, and hysterosacropexy refer to pelvic reconstructive procedures that involve fixation of pelvic organs to the sacrum, differing primarily in the anatomical structure suspended, namely the vagina, cervix, or uterus, while colpopexy denotes the broader concept of vaginal fixation. Vaginal procedures include sacrospinous ligament fixation (SSLF), uterosacral ligament suspension (USLS), McCall culdoplasty, the Manchester operation, and transvaginal mesh procedures, each associated with distinct risk profiles and compartment-specific recurrence patterns. In women who do not desire preservation of vaginal sexual function, obliterative surgery such as Neugebauer–Le Fort colpocleisis remains an effective and durable option. Current international guidelines emphasize that the choice of surgical technique should be individualized, taking into account patient age, sexual activity, comorbidities, prior pelvic surgery, and the type of compartmental defects, as well as surgeon expertise and available resources [9,10,11].

Given the central role of apical support in pelvic floor function, surgical correction of apical prolapse has evolved significantly over the past several decades. Sacrocolpopexy—fixation of the vaginal apex to the sacrum—was first described in the mid-20th century, being introduced as an abdominal procedure designed to restore vaginal apex support by suspending the vaginal vault or cervix to the anterior longitudinal ligament using synthetic mesh [12]. This technique aimed to recreate the normal vaginal axis and length while providing durable anatomic support. A 5-year follow-up study evaluating the long-term results of laparoscopic sacrocolpopexy demonstrated that 83.8% had no prolapse. The total reoperation rate was 3.5%. Only 3% of patients had mesh erosion into the bladder with no cases of vaginal erosion [13]. Over time, sacrocolpopexy has become widely regarded as the gold standard surgical treatment for apical prolapse, particularly in sexually active women, due to its superior long-term anatomic outcomes and lower recurrence rates compared with native tissue vaginal repairs. The procedure has further evolved with the advent of minimally invasive approaches, including laparoscopic and robotic-assisted sacrocolpopexy, which have demonstrated comparable efficacy with reduced blood loss, shorter hospital stays, and faster recovery [14]. Despite concerns regarding mesh-related complications, sacrocolpopexy remains one of the most extensively studied and effective procedures for the management of apical defects when performed with appropriate patient selection and surgical expertise.

Here, we present a comprehensive overview of current knowledge on pelvic organ prolapse (POP), ranging from its molecular basis to emerging diagnostic approaches. We also describe the evolution of surgical management, from traditional abdominal procedures to laparoscopic techniques, as well as recent advances in robot-assisted sacrocolpopexy.

## 2. Materials and Methods

**Scheme 1:**
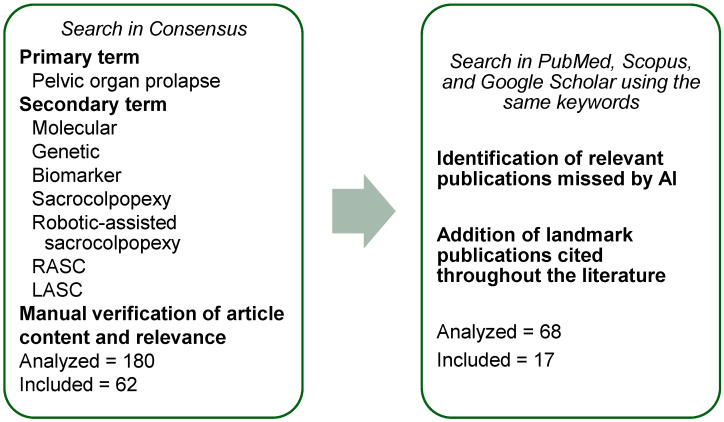
Flowchart of the literature search combining AI-based queries and database screening.

A literature search was conducted to identify peer-reviewed studies published up to February 2026. The initial search was performed using the Consensus AI-powered academic search engine (Boston, MA, USA), applying the primary term “pelvic organ prolapse” combined with the following keywords: “molecular,” “genetic,” “biomarker,” “sacrocolpopexy,” “robot-assisted sacrocolpopexy,” “RASC,” and “LASC.” Example queries included “pelvic organ prolapse genetic” and “pelvic organ prolapse RASC.” By default, Consensus returns the 20 most relevant articles per query. In total, 180 articles were manually screened for relevance and content, of which 62 publications were selected. The inclusion criteria were relevance to the subject, English language, and journal reputation. Journals ranked in the first and second quartiles (Q1/Q2) were prioritized, whereas Q3 journals were included only when no higher-ranked sources were available. Subsequently, additional searches were conducted in Scopus and Google Scholar using the same keywords to identify relevant publications not captured by the AI-based search and to include landmark studies cited across the literature. Results were ranked by citation count, and the 10 most cited publications were selected, excluding those already identified via Consensus. This process resulted in the evaluation of 23 additional articles, of which 12 were included based on relevance. Finally, a PubMed search was performed, screening the 10 most relevant records while excluding previously identified studies. This step resulted in the inclusion of 5 additional publications out of 45 screened. A flowchart illustrating the study selection process is presented in Scheme 1. Generally, articles were selected according to their scientific relevance and contribution to the field, with an effort to maintain a balance between landmark original studies, high-quality review articles, and the most recent advances. Priority was given to studies addressing pathophysiological mechanisms, diagnostic biomarkers, surgical outcomes, and innovations in minimally invasive and robotic surgical techniques.

## 3. Results

### 3.1. Molecular Insights into Apical Pelvic Organ Prolapse

Biomarkers, both genetic and protein-based, have significantly enhanced diagnostic accuracy and enabled earlier, often non-invasive detection of a wide range of diseases, particularly cancer [15]. Not surprisingly, numerous studies investigating the molecular basis of POP have been conducted to improve understanding of the disease and to identify relevant molecular markers. There are several reports on the heritability of POP. For example, it has been shown that POP segregates as a dominantly inherited trait with incomplete penetrance, exhibiting both maternal and paternal transmission, and confers an approximately fivefold increased risk to siblings of affected individuals compared with the general population [16]. A positive family history of pelvic organ prolapse is associated with an approximately 2.3–2.7-fold increased risk of developing POP and a 1.4-fold increased risk of recurrence [17]. In detailed genetic studies, no single mutation has been identified that is responsible for apical pelvic organ prolapse (POP). However, it has been established that variants of genes encoding proteins building connective tissue, muscle, and the extracellular matrix (ECM) can weaken the support of pelvic organs, increasing the risk of vaginal or uterine prolapse [18]. Several single-nucleotide polymorphisms (SNPs) in proteins associated with the remodeling of extracellular matrix were identified as either significant risk factors in POP or protective factors (Table 1). For example, the presence of SNP rs4870723 in collagen-related gene *COL14A1* has a strong correlation with occurrence of POP, while SNP rs56335679 in *COL18A1* can have potential protective properties [19]. Several studies have reported associations between POP and SNPs in genes involved in ECM processing, including rs370850 in *ADAMTS* family genes, rs3918254 in *MMP9*, and rs2277698 in *TIMP2* [20,21]. ADAMTS enzymes are involved in the organization of the ECM, while MMP9 mediates its degradation and TIMP2 regulates this process. Their balanced activity ensures optimal ECM stiffness, elasticity, and strength. SNPs rs2018736 in gene encoding *FBLN5*, a protein involved in the assembly of elastic fiber matrix, has also been linked to POP [22]. Above are only several examples of SNPs that can affect occurrence of POP, but more can be found in a recent review presenting the current state of the knowledge [18]. Since then, several new studies have been performed. For example, in 2025, candidate variants in ion channel genes and several others related to muscle and connective tissue with possible association with POP in young females with a family history have been identified [23]. The same group also identified three de novo missense variants in CSPG4, ITGA7, and MT-CO3 [24]. Alterations in those genes are known to cause dysfunctions in ECM, skeletal muscle, or mitochondria. The study, however, received some criticism regarding the exclusion of participants with a family history of POP, potentially confounding the obtained results [25]. Although the presence of a specific single-nucleotide polymorphism (SNP) within a given gene does not independently predispose to the development of pelvic organ prolapse (POP), it may contribute to the manifestation of the condition in women lacking classical risk factors such as pregnancy or obesity. Notably, the majority of identified SNPs were located within intronic regions, suggesting that they do not directly alter the protein sequence but may instead affect gene regulation or expression.

While the association of single-nucleotide polymorphisms (SNPs) with pelvic organ prolapse (POP) may offer potential diagnostic insight, alterations at the proteomic level must also be considered. Proteins constitute the functional machinery of both the cell and the extracellular matrix, directly influencing tissue structure and biomechanics. Comparative proteomic analysis of uterosacral ligament connective tissue obtained from women with POP and matched controls has been a subject of several studies. In the first study, Sun et al. found that galectin-1, transgelin, cyclophilin A, and cofilin-1 levels were decreased by more than two-fold [27]. In 2018, Li et al. reported that 88 proteins were differentially expressed, including 30 upregulated (1.2–1.66-fold change) and 58 downregulated (−1.21 to −1.78-fold change) proteins (examples in Table 2) [28]. Among the upregulated proteins were calponin-1 and calponin-3, which are involved in the regulation and modulation of smooth muscle contraction. Notably, many of the most downregulated proteins have unknown function; however, structural analysis suggested similarity to immunoglobulin-like domains have the potential to regulate cell–cell adhesion. Both studies examined the same tissue in postmenopausal women with stage III or higher pelvic organ prolapse (POP). Although there is partial functional overlap, particularly in proteins related to tissue structure, the specific proteins demonstrating altered expression differ between the studies. These findings suggest that POP represents a heterogeneous condition arising from multiple underlying molecular pathways.

**Figure 1:**
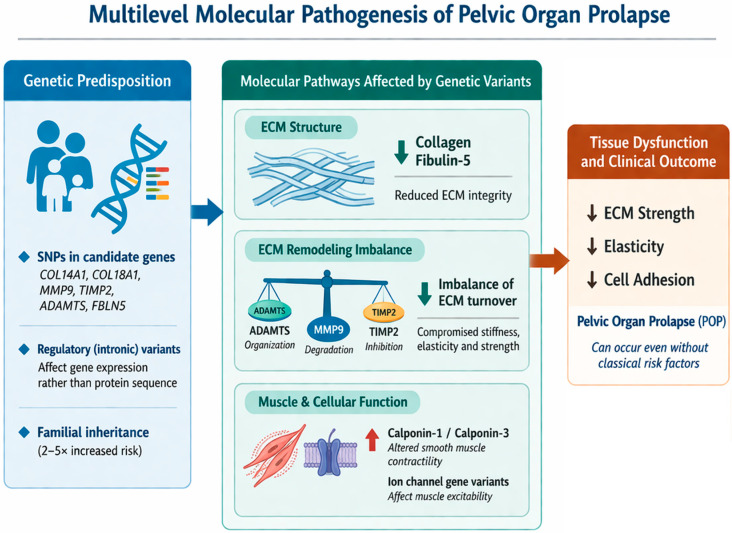
Multilevel molecular pathogenesis of pelvic organ prolapse (POP). Genetic predisposition to POP is polygenic and includes single-nucleotide polymorphisms (SNPs) in genes encoding extracellular matrix (ECM) components and regulators of ECM remodeling. Concurrent changes in smooth muscle function, including altered expression of contractile proteins such as calponins and ion channel–related pathways, further compromise pelvic floor support. Collectively, these molecular and cellular disturbances result in decreased tissue strength, elasticity, and cell adhesion, ultimately leading to pelvic organ prolapse, which may occur even in the absence of classical risk factors, such as parity.

POP can also be explored at the metabolomic level. Using capillary electrophoresis coupled with tandem mass spectrometry, Shama et al. found that, among 17 identified amino acids, methionine, glutamine, and histidine were significantly elevated in the pelvic connective tissue of affected patients (Table 3) [29]. In summary, POP does not have a single causation factor and can stem from multiple levels of molecular pathogenesis, from genetic mutations to ECM remodeling imbalance (Figure 1).

### 3.2. Biomarkers of the Pelvic Organ Prolapse

Although the molecular basis of pelvic organ prolapse (POP) has been increasingly elucidated, the next critical step is the development of screening assays capable of predicting both disease onset and severity. Currently, the diagnosis of pelvic organ prolapse (POP) is based on a comprehensive evaluation of clinical phenotype, medical history, and functional assessments. However, a number of assays have been developed. Wang et al. noticed that plasma levels of HSP10, ZC3H8, and UNC45A are significantly lower in patients with POP compared to healthy controls [30]. In a later study, Deng found that six metabolites: GPC, 1-methyladenosine, maleic acid, L-pyroglutamic acid, inosine, and citrate were significantly altered in both serum and urine samples from patients with POP. Receiver operating characteristic (ROC) curve analysis demonstrated that this metabolite panel discriminated patients with POP from controls with high accuracy [31].

With the increasing availability and declining cost of genetic testing, numerous studies have conducted genome-wide association analysis of pelvic organ prolapse (POP). Analysis of data from Icelandic cohorts and the UK Biobank identified eight sequence variants across seven loci associated with POP [32]. In another study, which analyzed genomic data from 28,086 individuals with POP and 546,291 controls of European ancestry, the first polygenic risk score for POP was developed. This score demonstrated predictive performance comparable to established clinical risk factors, such as number of deliveries [33].

Medical imaging can also be used to develop biomarkers for predicting POP risk and severity. Three-dimensional quantitative analysis of cardinal ligament using MRI demonstrated that volume, surface area, estimated mass, and maximum thickness were all significantly reduced in the POP in comparison to controls. This study also revealed that gross uterosacral ligament changes are not a universal feature of POP [34]. Recently, AI-enhanced 3D transperineal ultrasound has also been utilized. Laterolateral diameter showed significant correlations with POP severity across all conditions (rest, Valsalva, and contraction). Larger transverse dimensions were consistently associated with more advanced prolapse. Significant associations were observed for apical prolapse at rest and across dynamic conditions. In the anterior compartment, laterolateral diameter during Valsalva and contraction correlated with POP-Q points Aa and C, indicating its relevance as a parameter of pelvic support [35].

Unfortunately, there are no widely adopted clinical biomarkers that reliably predict who will develop prolapse or how rapidly it will progress. Given the existing infrastructure, imaging-derived biomarkers could likely be implemented most readily. However, these approaches are better suited for early detection and monitoring of disease progression rather than risk prediction. Polygenic risk factors may ultimately play a key role in risk estimation; however, their clinical utility will require validation in large, diverse cohorts representing different ancestries. In contrast, the detection of proteins or metabolites, due to the need for invasive sample collection, will likely remain primarily a research tool for elucidating the molecular basis of the disease, although it may also contribute to the development of nonsurgical therapeutic strategies.

### 3.3. Sacrocolpopexy in Pelvic Organ Prolapse Surgery: Epidemiology, Outcomes, and Determinants of Success

Currently, sacropexy (including colpo, cervico and hysteroscaropexy procedures) accounts for 3.3% of all POP surgical procedures in developed countries [36]. Of all POP surgeries, 54% were performed in the anterior compartment, while having 43% in the posterior and only 20% in the middle compartment. Total percentage exceeds 100% because some procedure descriptors include more than one POP procedure. Rates of prolapse interventions vary fivefold across OECD countries, with more than tenfold differences in specific POP procedures, highlighting major inconsistencies in care and the need for uniform surgical guidelines. In contrast, midurethral slings are consistently the standard procedure for female continence surgery across OECD countries [36].

In 2019, Ko and Lee presented criteria for success in POP surgery that can be adapted to establish the effectiveness of novel strategies for the improvement of surgical outcomes [37]. These include:

- Anatomically, the lowest point of descent is located above the hymenal plane.
- Normal bladder, bowel, and sexual function.
- Satisfactory quality of life.
- No complications are present.

The review also points to the fact that the success of POP repair largely depends on surgeon experience [37]. The 7th edition of Incontinence Report from 2023, prepared by the International Continence Society (ICS), summarizes 45 studies, summing up to 5584 laparoscopic sacrocolpopexies with their follow-up of more than 12 months [38]. The global success rate is 92%, with only 5% of reoperations stemming from either disease return (2.9%) or other complications (2.1%). Comprehensive data further support the safety profile of sacrocolpopexy [38]. Data presented from follow-up exceeding 12 months demonstrated a relatively low incidence of mesh-related and procedure-specific complications. Vaginal mesh exposure was reported in approximately 2% of cases. In comparison, mesh erosion after hysterosacropexy were observed in 1.1% [39]. Spondylodiscitis after sacrocolpopexy occurred in 0.9% of patients and was attributed primarily to iatrogenic disk injury caused by deep suture placement or the use of tackers or screws exceeding 5 mm in length during fixation at the sacral promontory [38]. Additionally, the incidence of de novo dyspareunia was low, reported in 2.3% of patients. These findings underscore the overall safety of sacrocolpopexy while highlighting the importance of meticulous surgical technique, particularly during promontory fixation, to minimize rare but serious complications.

Surgical success in the management of pelvic organ prolapse (POP) is multifactorial and extends beyond symptomatic improvement to include objective anatomical restoration and functional outcomes. From an anatomical perspective, success is commonly defined by postoperative positioning in which the lowest point of prolapse remains above the level of the hymen, a criterion widely used in both clinical practice and research settings [37]. However, different surgical techniques may alter vaginal axis orientation and influence compartment-specific recurrence patterns. Sacrospinous ligament fixation (SSLF) may result in posterior deviation of the vaginal axis, which has been hypothesized to contribute to dyspareunia in some women. It is also linked to anterior compartment prolapse recurrence in up to 12.6% of patients [40]. In contrast, procedures such as pectopexy and lateral suspension tend to restore vaginal axis more ventrally, which may reduce anterior compartment failure but can predispose to posterior compartment prolapse, including iatrogenic enterocele formation [41]. These observations highlight the importance of technique selection based on individual anatomical and functional considerations when defining and achieving surgical success.

Patient-reported quality-of-life (QoL) outcomes constitute a central component of success following surgical treatment for pelvic organ prolapse. In a recent systematic review by Guan and Han, encompassing 49 studies and a total of 945 patients, significant postoperative improvements in QoL were consistently demonstrated across different surgical techniques [42]. Most studies assessed outcomes using validated instruments such as the Pelvic Floor Distress Inventory-20 (PFDI-20), including its subscales: the Urinary Distress Inventory-6 (UDI-6), the Pelvic Organ Prolapse Distress Inventory-6 (POPDI-6), and the Colorectal-Anal Distress Inventory-8 (CRADI-8). The magnitude of QoL improvement varied according to the surgical approach, with sacrocolpopexy showing the greatest benefit, resulting in an average improvement of approximately 75%. This was followed by uterosacral ligament suspension (65%), transvaginal mesh procedures (60%), and sacrospinous ligament fixation (58%). These findings underscore the importance of incorporating patient-reported outcome measures into the evaluation of surgical efficacy and highlight all sacropexy procedures as most consistently associated with superior QoL improvement. Long-term patient-reported outcomes following laparoscopic sacrocolpopexy further support the durability and effectiveness of this procedure in the management of advanced pelvic organ prolapse. In a prospective cohort study by Pacquée et al., 331 patients undergoing laparoscopic sacrocolpopexy for symptomatic POP (POP-Q stage > 2) were followed for a mean duration of 85 months [43]. Assessment using the Patient Global Impression of Change (PGI-C) demonstrated sustained subjective improvement, with 84% of patients reporting a meaningful improvement in quality of life. Notably, persistent symptoms of prolapse were reported by only two patients (0.7%), indicating a very low rate of long-term symptomatic failure. These findings highlight the long-term efficacy and patient satisfaction associated with laparoscopic sacropexy. Comparative evidence further supports the effectiveness of sacropexy relative to sacrospinous ligament fixation. In a meta-analysis by Zhang et al., including 4120 patients undergoing either sacrocolpopexy or sacrospinous ligament fixation, both procedures demonstrated high overall success rates [44]. Sacrocolpopexy was associated with a slightly higher anatomical success rate compared with sacrospinous ligament fixation (91.45% vs. 88%) and a lower recurrence rate (8.3% vs. 11.5%). Importantly, the incidence of postoperative dyspareunia was significantly lower following sacrocolpopexy (4.6%) than after sacrospinous ligament fixation (14.3%). No statistically significant differences were observed between the two techniques with respect to the risk of intraoperative hemorrhage or postoperative gastrointestinal obstruction. These findings suggest that while both procedures are effective, sacrocolpopexy may offer advantages in terms of durability and sexual function outcomes.

Between 2022 and 2025, professional societies including the International Urogynecological Association (IUGA), American Urogynecologic Society (AUGS), European Urogynaecological Association (EUGA), and International Society for Gynecologic Endoscopy (ISGE) have issued various statements on the management of apical pelvic organ prolapse. Although no single universal guideline has replaced earlier recommendations, several key themes have emerged consistently across consensus statements, position papers, and guideline updates. Most notably, apical support should be addressed in the majority of prolapse repairs, as anterior or posterior repair without apical suspension is associated with higher failure rates. In addition, while sacrocolpopexy remains the benchmark for durability, there is no single ‘best’ procedure. Instead, surgical choice should be individualized based on factors such as age, comorbidity, sexual activity, preference for uterine preservation, risk of recurrence, and surgeon expertise. EUGA has recently supported the use of native tissue repair (NTR) as a first-line approach for pelvic organ prolapse (POP), while acknowledging that mesh-reinforced procedures may be appropriate in selected clinical scenarios [45]. In 2025, ISGE emphasized tailoring surgical techniques to prolapse severity to reduce the risk of post-hysterectomy vault prolapse, thereby improving outcomes and patient quality of life [46].

### 3.4. Possible Complications and Ways to Avoid Them

While sacrocolpopexy is increasingly employed as a standard surgical approach for the management of apical pelvic organ prolapse, and ongoing technical refinements and advances in surgical materials have contributed to improved safety profiles, the procedure continues to be associated with specific anatomical and operative risks. A thorough understanding of these potential hazards remains essential for the surgeon. In particular, there are three principal anatomical domains that represent major sources of perioperative and postoperative complications: vascular structures, the ureters, and the pelvic autonomic nerves. Injury to these structures may occur during dissection, mesh placement, or fixation and can result in significant morbidity if not promptly recognized and appropriately managed [47].

Vascular injury is among the most serious and potentially life-threatening complications of all types of sacropexy procedures, particularly during dissection and mesh fixation at the level of the sacral promontory. Early reports described vascular complication rates of up to 4.4%, with rare cases of procedure-related mortality attributed to major hemorrhage [48]. The vessels most frequently at risk include the median sacral vessels, which often demonstrate anatomical variability and accessory branches, as well as the left common iliac vein, the right common iliac artery, and the presacral venous plexus, all of which are located in close proximity to the surgical field [49]. Improved understanding of sacral promontory anatomy, along with advances in minimally invasive techniques and surgeon experience, has substantially reduced the incidence and severity of vascular complications. In contemporary series, mean intraoperative blood loss during sacrocolpopexy is reported to be low and comparable to that observed in laparoscopic lateral suspension procedures, averaging at approximately 100 mL [50]. Particular attention should be paid to the close proximity of major vascular structures to the midline at the level of the sacral promontory. The left common iliac vein may be located as close as 9 mm from the midline, while the right common iliac artery may lie only 14 mm from the midline, substantially increasing the risk of vascular injury, especially in obese patients and in those with adhesions from prior abdominal or pelvic surgery. In addition, the presacral venous plexuses may be situated as little as 4 mm below the sacral promontory, placing them at significant risk during deep dissection or when the implant is fixed at lower sacral levels, particularly at S2–S3.

Ureteral injury remains a recognized complication of colpo, cervico and hysterosacropexy, with the right ureter being particularly vulnerable during dissection in the presacral and pararectal regions. Anatomical studies have demonstrated that the right ureter may course as close as 13 mm from the midline, placing it at risk during preparation of the anterior longitudinal ligament and during retroperitoneal tunneling for mesh placement. The reported incidence of ureteral injury during colpo, cervico and hysterosacropexy is approximately 1%, with increased risk associated with limited visualization, altered anatomy, or technically demanding dissections. Given this close anatomical relationship and the potential for significant morbidity associated with delayed recognition of ureteral damage, routine identification and clear visualization of the ureter during critical surgical steps are strongly recommended to minimize the risk of injury [47].

Nerve injury is an infrequent but clinically significant complication of sacrocolpopexy, primarily related to dissection and mesh fixation in close proximity to the sacral promontory. The sacral nerve roots may be injured when mesh fixation is performed at lower sacral levels, particularly at S2–S3, potentially resulting in sensory or motor disturbances. In addition, injury to the inferior hypogastric plexus has been associated with postoperative functional disorders, including de novo overactive bladder symptoms, defecatory dysfunction such as constipation, and sexual dysfunction. Although the overall reported risk of nerve injury remains low, estimated at less than 5%, these complications may have a substantial impact on quality of life. Careful identification of neural structures and the adoption of nerve-sparing surgical techniques are therefore essential to minimize neurological morbidity during sacrocolpopexy [51,52]. Meticulous surgical technique, precise anatomical knowledge, and heightened intraoperative vigilance are critical to minimizing these risks and optimizing patient outcomes.

It should be noted that laparoscopic lateral suspension (LLS), including the Dubuisson technique, has been proposed as an alternative approach for the surgical management of pelvic organ prolapse that avoids dissection and fixation at the sacral promontory. By eliminating promontory exposure, this technique minimizes the risk of vascular and nerve injury associated with presacral dissection. According to a systematic review by Campagna et al., which analyzed 13 studies encompassing 1066 patients undergoing LLS, the procedure demonstrated favorable anatomical and functional outcomes [53]. Reported anatomical success rates reached approximately 90%, while subjective success ranged from 74% to 100%. Major postoperative complications (Clavien–Dindo grade ≥ 3) were uncommon, occurring in approximately 1% of cases, and mesh erosion rates varied between 0% and 13%. Despite these advantages, laparoscopic lateral suspension has been associated with a potential predisposition to iatrogenic enterocele, underscoring the need for careful patient selection and surgical technique refinement.

Many countries have restricted or prohibited the use of transvaginal meshes due to an unfavorable risk–benefit profile and a high incidence of mesh-related complications [38]. On 16 April 2019, the FDA ordered a halt to the sale of surgical mesh intended for transvaginal repair of pelvic organ prolapse. The FDA, however, continued to allow the use of mesh in abdominal, laparoscopic, and robotic sacrocolpopexy, as evidence suggests lower mesh exposure rates, more durable long-term apical support, and a more favorable benefit–risk profile compared with transvaginal mesh repairs [54]. It should be noted that despite this clear distinction, 71% of news media articles did not identify or clarify that the FDA ban applied to only transvaginal mesh [55]. This has negatively affected patient perceptions and their willingness to undergo procedures involving the mesh.

### 3.5. Evolution of the Technique

#### 3.5.1. Abdominal Sacrocolpopexy

After its development sacrocolpopexy has become widely adopted as a treatment for pelvic organ prolapse. A systematic review of the published literature (1966–2004) evaluating abdominal sacrocolpopexy identified substantial heterogeneity in reported outcomes and follow-up intervals, precluding meta-analysis. Across studies with follow-up ranging from 6 months to 3 years, anatomical success rates were high, with absence of apical prolapse reported in 78–100% of cases and absence of any postoperative prolapse in 58–100%. Median reoperation rates were 4.4% for recurrent prolapse. The overall mesh erosion rate was 3.4%, with inconsistent evidence regarding increased risk when concomitant total hysterectomy was performed. Small bowel obstruction requiring surgery occurred in a median of 1.1% of cases [48]. Reports specifically describing the abdominal approach have become increasingly scarce, with very few recent case reports identified. This shift in the literature reflects the broader evolution of pelvic organ prolapse surgery, where the development of abdominal sacropexy has largely plateaued, while technological and procedural advancements have increasingly concentrated on laparoscopic and robotic-assisted techniques.

#### 3.5.2. From Abdominal to Laparoscopic

In one of the first reviews of the laparoscopic procedure, it was already visible that it offers great advantages over abdominal surgery, including faster recovery and improved visualization. This retrospective study evaluated the feasibility and early outcomes of laparoscopic sacrocolpopexy with concomitant procedures in 77 women with symptomatic uterovaginal prolapse. The authors report that the purely laparoscopic approach was technically feasible after initial experience, with operative times decreasing significantly as proficiency improved. However, it should be noted that operative time and postoperative complications are related to the surgeon’s experience but remain comparable to those found in laparotomy. Conversion to laparotomy was required in 6 of 83 attempted cases, and major intraoperative complications were uncommon (2 bladder injuries and 1 rectal injury). Short-term postoperative morbidity included urinary tract infection, unexplained fever, and hematomas, with three patients requiring reoperation. At a mean follow-up of approximately 11 months, most patients demonstrated satisfactory anatomic outcomes, though a small number experienced recurrent prolapse [56]. It should be stated that this early procedure was performed with dual strips of synthetic mesh, in contrast to the current single strip mesh technique.

Further analysis of three RCTs (randomized controlled trials) of 247 patients demonstrated no differences in apical defect recurrence between laparoscopic and abdominal sacrocolpopexy techniques [57]. A large retrospective cohort study evaluated 660 patients who underwent laparoscopic sacrocolpopexy for post-hysterectomy vault prolapse between 2005 and 2017, with a median follow-up of over four years. Laparoscopic sacrocolpopexy was associated with high satisfaction and low reoperation rates. Vaginal mesh exposure occurred in 0.7% of cases, with most managed conservatively and a small proportion requiring surgical excision. Non-absorbable vaginal suture erosion was observed in 0.6%, similarly managed either conservatively or with minor surgical intervention. These findings support laparoscopic sacrocolpopexy as a safe and effective option for apical prolapse, with a low rate of mesh exposure and favorable anatomical outcomes [58].

#### 3.5.3. Refinement of Laparoscopic Surgical Techniques

Recently, Campagna and colleagues conducted a multicenter retrospective cohort study comparing laparoscopic sacral hysteropexy (LSHP) with laparoscopic sacral colpopexy combined with supracervical hysterectomy (LSCP/SCH) for symptomatic pelvic organ prolapse. Their findings demonstrated comparable anatomical and subjective success rates at 24 months between the uterus-preserving LSHP and the LSCP/SCH approaches, with no statistically significant differences in objective cure or patient satisfaction. Notably, LSHP was associated with a shorter median operative time, while both procedures exhibited similar profiles in estimated blood loss, conversion to laparotomy, and perioperative complications. These results support LSHP as a safe and effective alternative to sacropexy with hysterectomy for women desiring uterine preservation, without compromising mid-term outcomes [59]. A subsequent study comparing LSHP and LSCP demonstrated that, at 24 months of follow-up, composite failure rates were 10.7% in the supracervical hysterectomy group and 3.6% in the uterine preservation group [60]. Conclusions similar to the Campagna report [59] were reached by Ruffolo et al., who compared hysterectomy and hysteropexy in vaginal native tissue repair for pelvic organ prolapse. However, hysterectomy was associated with longer operative time, greater blood loss, and a longer hospital stay [61]. In another study, Brennand et al. reported that, at 1 year, uterine-preserving surgery was associated with a lower risk of composite recurrence [62].

In their comparative study published in the World Journal of Gastrointestinal Endoscopy, Cosma et al. evaluated postoperative bowel function following nerve-preserving versus standard laparoscopic sacropexy for apical pelvic organ prolapse. The authors investigated whether careful identification and preservation of the hypogastric nerves during promontory dissection could reduce functional gastrointestinal sequelae commonly attributed to autonomic injury. Their analysis demonstrated a significantly lower incidence of de novo postoperative constipation and obstructed defecation symptoms in the nerve-sparing group compared with the standard technique, without a concomitant increase in operative time. Importantly, the nerve-sparing approach did not adversely affect apical recurrence rates or the incidence of late postoperative complications when compared with the conventional procedure. These findings provide clinical evidence supporting the functional advantages of autonomic nerve preservation during sacropexy and reinforce the importance of meticulous presacral dissection to minimize iatrogenic pelvic denervation [63]. However, a recent review highlights that, despite these developments, considerable heterogeneity remains among surgeons regarding operative technique and the use of concomitant procedures during sacrocolpopexy, which complicates direct comparison of the reported technical advancements [64].

#### 3.5.4. Moving Beyond Laparoscopy to Robot-Assisted Sacrocolpopexy: Technological Progress at Increased Cost but Shorter Learning Curve

Robot-assisted surgery was first introduced into clinical practice in 1985 with the use of the PUMA 560 system for neurosurgical biopsy. As of 2023, over 11 million robotic procedures were performed worldwide, solely with the use of the da Vinci system [65]. A recent systematic review of 45 randomized controlled trials encompassing 7364 patients across urologic, gynecologic, and general surgery procedures found that robotic and conventional laparoscopic approaches yield broadly comparable clinical outcomes in minimally invasive abdominal and pelvic surgery. No significant differences were observed in mortality, overall complication rates, length of hospital stay, conversion to open surgery, or long-term oncologic outcomes in the majority of trials. Robotic surgery was consistently associated with longer operative times and significantly higher total procedural costs, while potential benefits in selected studies included improved patient-reported outcomes, cosmetic satisfaction, and procedure-specific functional recovery. However, heterogeneity in study design, procedural complexity, and reporting standards, as well as limited long-term and quality-of-life data, constrain definitive conclusions regarding clinical superiority [66]. Raimondo et al. show that for radical hysterectomy, bilateral salpingo-oophorectomy, and lymph node dissection, the minimum number of procedures required to reach the learning curve was smaller in robotic-assisted laparoscopy than laparoscopic surgery [67]. A systematic review by Grassini et al. evaluated the role of robotic surgery (RS) in benign gynecologic pathology, including hysterectomy, myomectomy, endometriosis surgery, and pelvic organ prolapse repair. The review included 22 studies (12 randomized controlled trials and 10 retrospective cohort studies) comprising 269,728 patients. In procedures such as hysterectomy and myomectomy, RS showed similar perioperative outcomes, complication rates, and recovery profiles, although operative times and healthcare costs were often higher with robotic systems. Similarly, robotic sacrocolpopexy possessed comparable clinical efficacy to laparoscopic approaches. The authors conclude that for complex conditions, such as deep infiltrating endometriosis, robotic platforms may provide technical advantages due to improved three-dimensional visualization and enhanced instrument dexterity [68]. In a recent single-center study, Lallemant et al. compared laparoscopic sacrocolpopexy with robotic-assisted sacrocolpopexy. The reoperation rate for recurrent POP was higher in the robotic-assisted group (9.2% vs. 1.2% for the laparoscopic approach) [69]. No significant differences were found in intraoperative or postoperative complications. Callewaert et al. conducted a systematic review comparing laparoscopic sacrocolpopexy (LSC) and robotic-assisted sacrocolpopexy (RASC) for the treatment of pelvic organ prolapse. Database searches identified two randomized controlled trials (*n* = 78) evaluating clinical outcomes and healthcare costs. Operative time results were inconsistent between studies, with one trial reporting shorter operative times for LSC and the other demonstrating no significant difference. Postoperative pain was reported to be slightly higher following robotic surgery in the short term, although the clinical relevance appeared limited. Both trials consistently showed that RASC was associated with significantly higher costs, primarily due to the acquisition, maintenance, and instrument expenses of robotic systems, without any improvement in clinical outcomes [70]. The most recent cost comparison of abdominal, laparoscopic, and robot-assisted sacrocolpopexy reported median costs of $32,657, $42,273, and $52,389, respectively. Although the abdominal approach was the least costly, it was associated with more severe short-term complications [71]. It should be noted, however, that ongoing developments in robotics, together with increased market competition resulting from the entry of new companies, are expected to drive prices down. A recent study comparing the Senhance and da Vinci platforms in sacrocolpopexy demonstrated that, although both platforms yielded similar outcomes, complication rates, and overall costs, adjusted costs (e.g., accounting for operative time) were more favorable for the Senhance system [72]. While cost-effectiveness is an important consideration, patient characteristics and medical history, as well as surgeon experience, should also be taken into account. Strauss et al. reported that, in laparoscopic sacrocolpopexy, higher BMI, parity, and age were associated with longer operative time and postoperative hospital stay, whereas these factors did not significantly influence time-related outcomes in robotic-assisted procedures [73]. Additionally, Dehan et al. reported that up to three RASC procedures could be performed per day, compared with two LSC procedures, suggesting a potential improvement in operating room efficiency [74].

Further development in robotic sacrocolpopexy was recently reported in a single-center randomized controlled trial by Matanes and colleagues. The authors compared robotic single-site sacrocolpopexy with multi-port robotic sacrocolpopexy for the surgical management of apical compartment prolapse in women with POP-Q stages 2–4. The study found that the single-site approach was associated with longer operative and console times compared with the multi-port technique, without demonstrable improvements in key clinical outcomes such as estimated blood loss, intraoperative or postoperative complications, or quality-of-life measures at short-term follow-up. Both approaches yielded comparable anatomical repair and perioperative safety profiles. Patient-reported assessments of scars were more favorable with the single-site technique; however, the increased operative time may reflect technical challenges inherent in the single-site approach. These findings suggest that while robotic single-site sacrocolpopexy is a feasible and safe minimally invasive option for apical prolapse, it does not confer clear perioperative or functional advantages over the conventional multi-port robotic technique [75].

As presented above, current knowledge regarding laparoscopic and robot-assisted sacropexy highlights that both conventional laparoscopic sacrocolpopexy and emerging robot-assisted approaches offer the advantages of minimally invasive surgery—reduced blood loss, shorter hospital stay, and faster recovery—while achieving high anatomical success rates comparable to open abdominal procedures. Robot-assisted sacrocolpopexy, facilitated by enhanced visualization and instrument dexterity, may further mitigate the technical challenges of deep pelvic dissection and suturing that limit widespread adoption of purely laparoscopic techniques, although it incurs greater operative costs and requires specific surgical training [76].

**Figure 2:**
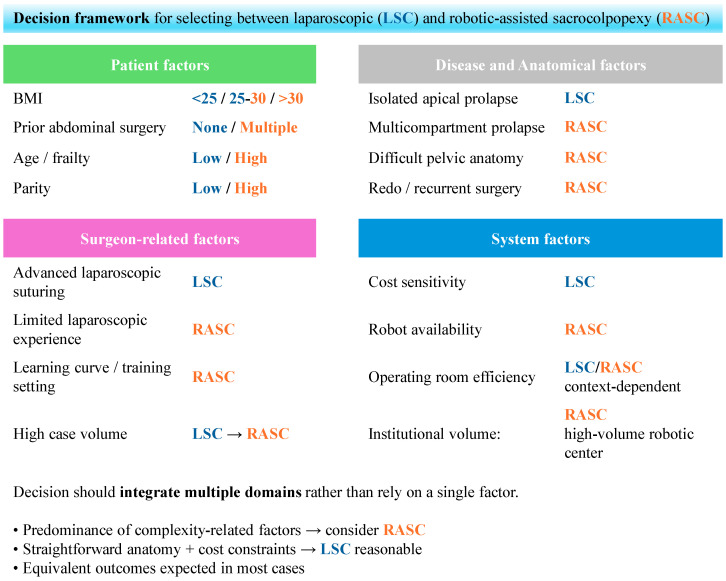
Multidimensional decision framework for selecting between laparoscopic (LSC) and robotic-assisted sacrocolpopexy (RASC). The choice is influenced by patient characteristics, anatomical complexity, surgeon experience, and system-level factors.

As presented above, while cost has been the major factor in the LSC and RASC comparison, there are a number of other factors that have to be considered when deciding between the two methods, which we summarized in Figure 2. A brief numerical comparison between various sacrocolpopexy approaches for the treatment of middle compartment POP is given in Table 4.

### 3.6. Own Experience of the Introduction of a Sacropexy into Clinical Setting

While there are multiple statistical data on success rates of various POP surgeries and novel approaches, a hands-on experience of introducing this method is rarely presented in the literature. Therefore, we would like to share our experience of introduction of a sacropexy (including colpo, cervico and hysteropexy) into clinical setting. After completing intense theoretical and practical training in a urogynecological reference center—Department of Gynecology and Obstetrics CM UKSW, Międzyleski Szpital Specjalistyczny, Warsaw, Poland (Head: Prof. Ewa Barcz)—the surgical team from the Department of Obstetrics and Gynecology, Wroclaw Medical University, from November 2024 to March 2026, successfully performed 75 laparoscopic sacropexies. Adapting the key learning points established by Claerhout et al., our learning curve reached a plateau for a group of surgeons after 30 operations [79].

## 4. Conclusions

Over the past three decades, minimally invasive abdominal approaches have become increasingly prominent in the surgical management of pelvic organ prolapse, combining the lower morbidity of vaginal surgery with the durable anatomical outcomes of open abdominal repair. Research into the molecular basis of the disease has revealed a complex network of interdependencies spanning genomic, proteomic, and metabolomic levels of cellular function. The summarized results, however, only partially overlap in their proposed targets, highlighting the need for further research using a multi-omics approach on the same samples. While these advances are crucial for understanding human physiology, they have not yet been translated into clear pathways for novel therapeutic strategies. Several potential biomarkers have been proposed. At present, non-invasive, imaging-derived biomarkers, particularly those enhanced by artificial intelligence, are arguably closest to clinical implementation. However, they are best suited for disease staging and monitoring. In the future, polygenic risk factors will likely become more widely used, as whole-genome sequencing becomes cheaper each year and a single diagnostic test can provide a multitude of information at once, avoiding the single test–consultation loop. Ideally, the integration of multi-omics data with clinical and imaging parameters will be required to develop robust, clinically applicable predictive models. This highlights the need for closer cooperation between molecular biologists and clinical practitioners. Sacrocolpopexy remains the gold standard procedure for apical vaginal suspension, and although conventional laparoscopy was introduced in the 1990s, widespread adoption was initially limited by technical complexity, particularly laparoscopic suturing. The introduction of robotic-assisted platforms following regulatory approval for gynecologic surgery facilitated broader uptake of minimally invasive sacrocolpopexy by enhancing visualization, dexterity, and technical accessibility for surgeons, especially in complex cases.

## Figures and Tables

**Table 1:** Examples of genes in which single-nucleotide polymorphisms (SNPs) have been associated with the development of pelvic organ prolapse. Notably, the majority of the identified SNPs are located within intronic regions. These variants do not directly modify the amino acid sequence of the encoded proteins; rather, they may influence gene regulation, transcriptional activity, or other mechanisms controlling gene expression.

Gene	Protein Name	SNP Identification	Molecular Function [26]	Ref.
*COL14A1*	Collagen alpha-1(XIV) chain	rs4870723	Adhesive role by integrating collagen bundles	[19]
*ADAMTS1*	A disintegrin and metalloproteinase with thrombospondin motifs 1	rs370850	Metalloprotease which cleaves aggrecan	[20]
*TIMP2*	Metalloproteinase inhibitor 2	rs2277698	Inhibitor of metalloproteinases (MMPs)	[20]
*MMP9*	Matrix metalloproteinase-9	rs3918254	Local proteolysis of the extracellular matrix	[21]
*FBLN5*	Fibulin-5	rs2018736	Essential for elastic fiber formation	[22]
*ATP1A4*	Sodium/potassium-transporting ATPase subunit alpha-4	rs779877987	Catalyzes the hydrolysis of ATP coupled with the exchange of sodium and potassium ions across the plasma membrane	[23]

**Table 2:** Examples of proteins exhibiting altered expression levels in pelvic organ prolapse affected tissues. Each study identified multiple upregulated and downregulated proteins; here, only those with the greatest magnitude of change are presented.

Protein Name	Status in POP	Molecular Function [26]	Ref.
Galectin-1	Downregulated	Binds β-galactosides and complex carbohydrates; regulates apoptosis, proliferation, and differentiation.	[27]
Calponin-1	Upregulated	Thin filament-associated protein involved in regulation of smooth muscle contraction.	[28]
VH3 protein	Downregulated	No known function.	[28]

**Table 4:** Brief comparison between various sacropexy approaches for treatment of middle-compartment POP. Objective success was judged by a physical examination or imaging, using predefined anatomical criteria.

Approach and Typically Reviewed Period	Typical Anatomic Success (Objective)	Common Complications	Example References
Open abdominal sacrocolpopexy	78–100% apical 58–100% other compartment	Mesh erosion 3.4% SBO surgery 1.1% POP reoperation 4.4%	[48]
Laparoscopic sacrocolpopexy	90–91% objective (all compartments)	Mesh erosion 2–3% POP reoperation 6%	[77]
		Mesh erosion 0–13%	[53]
Robotic-assisted sacrocolpopexy	98.6% apical84–100% other compartment	Mesh erosion 2–4% Serious postoperative complications 2%	[78]

**Table 3:** Metabolites exhibiting altered levels in pelvic organ prolapse-affected tissues.

Protein Name	Status in POP	Ref.
Methionine	Significantly increased	[29]
Glutamine	Significantly increased	[29]
Histidine	Significantly increased	[29]

## Data Availability

No new data were created or analyzed in this study. Data sharing is not applicable to this article.

## Abbreviations

The following abbreviations are used in this manuscript:

CRADI-8	Colorectal-Anal Distress Inventory-8
ECM	Extracellular Matrix
EUGA	European Urogynaecological Association
ICS	International Continence Society
IUGA	International Urogynecological Association
LASC	Laparoscopic Sacrocolpopexy
LLS	Laparoscopic Lateral Suspension
LSHP	Laparoscopic Sacral Hysteropexy
MMP	Matrix metalloproteinase
MRI	Magnetic Resonance Imaging
OECD	Organization for Economic Cooperation and Development
PFDI-20	Pelvic Floor Distress Inventory-20
PGI-C	Patient Global Impression of Change
POPDI-6	Pelvic Organ Prolapse Distress Inventory-6
POP	Pelvic Organ Prolapse
POP-Q	Pelvic Organ Prolapse Quantification system
QoL	Quality of Life
RASC	Robotic-Assisted Sacrocolpopexy
RCT	Randomized Controlled Trial
ROC	Receiver Operating Characteristic
SBO	Small Bowel Obstruction
SNP	Single-Nucleotide Polymorphism
SSLF	Sacrospinous Ligament Fixation
UDI-6	Urinary Distress Inventory-6
USLS	Uterosacral Ligament Suspension
